# Persistent and fatal severe acute respiratory syndrome coronavirus 2 infection in a patient with severe hypogammaglobulinemia: a case report

**DOI:** 10.1186/s13256-023-03917-x

**Published:** 2023-05-13

**Authors:** Vanessa Bedoya-Joaqui, María I. Gutiérrez-López, Paola A. Caicedo, María F. Villegas-Torres, Ludwig L. Albornoz-Tovar, Juan D. Vélez, Alejandra Hidalgo-Cardona, Gabriel J. Tobón, Carlos A. Cañas

**Affiliations:** 1 Departamento de Medicina Interna, Unidad de Reumatología, Universidad Icesi, Facultad de Ciencias de la Salud, 760031 Cali, Colombia; 2 Departamento de Biología, Universidad Icesi, Facultad de Ciencias de la Salud, 760031 Cali, Colombia; 3 Centro de Investigaciones Microbiológicas, Departamento de Ciencias Biológicas, Universidad de los Andes, 111711 Bogotá, Colombia; 4 Departamento de Patología, Fundación Valle del Lili, 760032 Cali, Colombia; 5 Unidad de Infectología, Fundación Valle del Lili, 760032 Cali, Colombia; 6 Centro de Investigaciones Clínicas, Fundación Valle del Lili, 760032 Cali, Colombia; 7 Department of Medical Microbiology, Immunology and Cell Biology, Southern Illinois University School of Medicine, Springfield, IL 62794-9620 USA; 8 Unidad de Reumatología, Fundación Valle del Lili, Cra.98 No. 18-49, 760032 Cali, Colombia; 9 CIRAT: Centro de Investigación en Reumatología, Autoinmunidad y Medicina Traslacional, Universidad Icesi, 760031 Cali, Colombia

**Keywords:** COVID-19, Immunodeficiency, Mutations, Variants

## Abstract

**Background:**

Viruses are constantly changing as a result of mutations, and new viral variants are expected to appear over time. The virus that causes coronavirus disease 2019, severe acute respiratory syndrome coronavirus 2, is not excluded from this condition. Patients with some types of immunodeficiency have been reported to experience symptoms that vary from mild to severe, or even death, after being infected with severe acute respiratory syndrome coronavirus 2. We report a case of a woman with severe hypogammaglobulinemia who developed a prolonged and fatal severe acute respiratory syndrome coronavirus 2 infection.

**Case presentation:**

A 60-year-old mestizo female with a previous history of severe hypogammaglobulinemia manifested by recurrent pulmonary infections and follicular bronchiolitis. She received a monthly treatment of intravenous immunoglobulins and was admitted after report of a neurological manifestation related to a left thalamic inflammatory lesion, for a duration of 2 weeks of hospitalization, indicated for the study of her neurological condition, including brain biopsy. Both on admission and 1 week later, nasopharyngeal polymerase chain reaction tests for severe acute respiratory syndrome coronavirus 2 were performed and reported negative. In the third week of hospitalization, she developed pulmonary symptoms, and a positive test result for severe acute respiratory syndrome coronavirus 2 was evidenced. On Day 3, the patients’ condition worsened as the infection progressed to respiratory failure and required mechanical ventilation. On Day 8 after the coronavirus disease 2019 diagnosis, the polymerase chain reaction test for severe acute respiratory syndrome coronavirus 2 showed persistent detection of the virus. Various bacterial coinfections, including *Klebsiella pneumoniae* and *Enterobacter cloacae*, were diagnosed and treated. On Day 35, her pulmonary symptoms worsened, and the results of the severe acute respiratory syndrome coronavirus 2 polymerase chain reaction test remained positive. On Day 36, despite all the respiratory support, the patient died. The severe acute respiratory syndrome coronavirus 2 virus was sequenced at the beginning and 8 days after the onset of the disease, and the strain, without obvious mutations in the gene that encodes spike protein, was identified.

**Conclusions:**

This clinical case showed persistent severe acute respiratory syndrome coronavirus 2 detection after 35 days of infection in a patient with severe hypogammaglobulinemia. The sequencing of the virus showed no mutations on the spike protein at 8 days, indicating that, in this case, the persistence of the viral detection was associated with immunodeficiency instead of changes in the viral components.

## Background

Viruses are constantly changing as a result of mutations, and new viral variants are expected to appear over time. The virus that causes coronavirus disease 2019 (COVID-19), severe acute respiratory syndrome coronavirus 2 (SARS-CoV-2), is not excluded from this condition. Several groups of scientists worldwide permanently monitor the changes in this virus, based mainly on its genetic analysis.

Patients with some types of immunodeficiency have been reported to experience symptoms that vary from mild to severe, or even death, after being infected with SARS-CoV-2. Patients with comorbidities are the most susceptible for developing severe, chronic, or recurrent types of COVID-19 lung infection [1]. Furthermore, it increases the risk of mutations and new viral variants [2]. Some variants spread more easily and quickly than others, which would allow for an increased number of COVID-19 cases in a specific period of time. Increases in the number of cases will increase the burden on healthcare resources and could mean more hospitalizations and deaths.

On the other hand, coinfection is one of the risk factors for SARS-CoV-2-related lung infection in patients with immunodeficiencies. There are a variety of synergistic biological interactions between viruses and bacteria, which have led to an increased risk of bacterial infections (where a primary viral infection is present) and vice versa.

Bacteria and other pathogens have been shown to complicate viral pneumonia with poor patient outcomes [3].

We report a case of a woman with severe hypogammaglobulinemia who developed a prolonged and fatal SARS-CoV-2 infection without showing, at least in the first 2 weeks of her clinical course, mutations in the spike (S) protein gene of the virus. The patient presented several bacterial coinfections that contributed to her lung deterioration.

## Case presentation

The patient was a 60-year-old mestizo female with severe hypogammaglobulinemia since the age of 55 years complicated by recurrent pulmonary infections and follicular bronchiolitis. The patient did not report any other relevant history such as diabetes mellitus, arterial hypertension, human immunodeficiency virus (HIV) infection, surgery, allergies, or autoimmune disease, neoplastic disease, drugs, or other important conditions. Single gene or other genetic defects associated with immunodeficiencies were not studied. She had no history of smoking, alcohol, or illicit drug use. She had two pregnancies that came to term without complications. Her menopause began at 51 years of age. She did not report having relatives with immunodeficient states or other important pathologies. She received monthly treatments of intravenous immunoglobulins with good control of symptoms and without development of new infections in the last 2 years. She had received the Pfizer vaccine for Covid-19 10 months and 4 months before admission. She did not report having had COVID-19. Four months before admission, intravenous immune globulin (IVIG) was not applied due to lack of availability of the drug in our country (Colombia).

One month before admission, the patient began to report progressive headaches, a feeling of dizziness, and weakness. She consulted a neurologist who found no relevant findings on the general physical and neurological examination. He decided to perform a brain magnetic resonance imaging (MRI) scan where he found a left thalamic lesion with inflammatory characteristics. The neurologist referred the patient for hospitalization.

On admission, the medical history revealed no other symptoms. In her physical examination, nothing pathological was found. Her vital signs, cardiopulmonary and neurological examinations, skin, abdomen, and extremities were all normal.

During hospitalization, various analyses were made to rule out infectious processes that include toxoplasma, cytomegalovirus, syphilis, HIV, cryptococcosis, histoplasmosis, and tuberculosis, among others, as well as autoimmune or neoplastic conditions (based on serological and cerebrospinal fluid studies). Antinuclear antibodies and antineutrophil cytoplasmic antibodies were negative, and complement levels were normal. Her blood count revealed: hemoglobin (Hb) 10.2 g/L, white blood cell count 4054/mm^3^, neutrophils 3750/mm^3^, lymphocytes 252/mm^3^, monocytes 52/mm^3^, and platelets 212.00/mm^3^. CD4^+^ T lymphocytes were 122/mm^3^. Immunoglobulin (Ig)G was 110 mg/dL, IgM was 56.7 mg/dL), and IgA was 105 mg/dL. Liver and kidney function tests were normal. Anti-SARS-CoV-2 IgM and IgG antibodies were negative. Both on admission and 1 week later, nasopharyngeal polymerase chain reaction (PCR) tests for SARS-CoV-2 were performed and reported negative.

Fourteen days after admission, a brain MRI was repeated, and the thalamic lesion had increased in size. In the absence of a diagnosis, it was decided to carry out a brain biopsy (which revealed non-specific lymphoplasmacytic infiltrate. Neoplasms, infections, and vasculitis were ruled out). Testing for single-gene and other genetic defects testing associated with immunodeficiencies was not carried out. Three days after, the patient developed respiratory distress, cough, and fever. Her oxygen saturation (SpO_2_) level was 56% on room air. A PCR test for SARS-CoV-2 showed a positive result, as the virus was detected in a nasopharyngeal swab sample (Day 1). Computed tomography (CT) scan of the chest revealed a typical and severe viral pneumonia associated with COVID-19 with diffuse interstitial infiltrates.

**Fig. 1:**
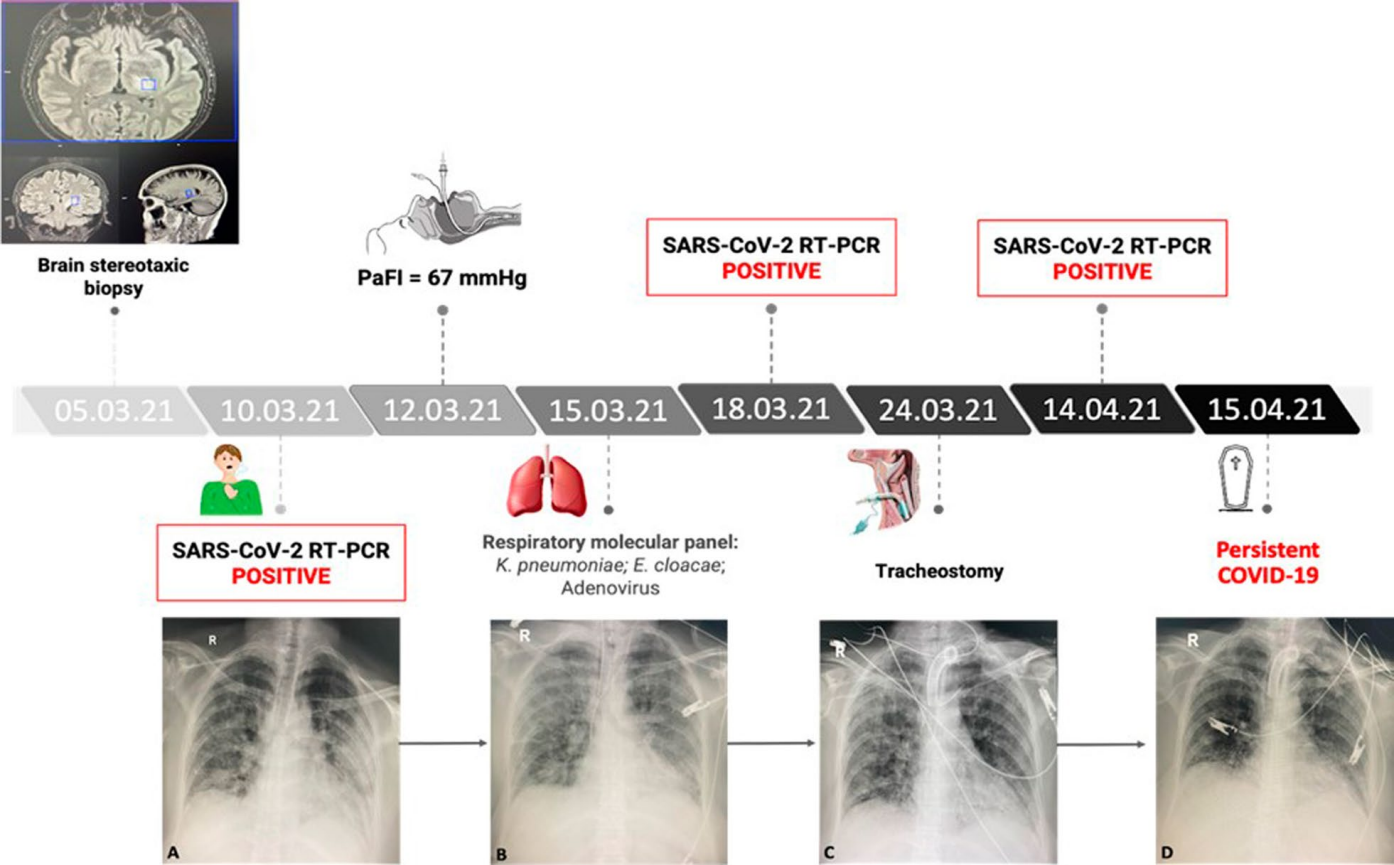
Timeline of persistent COVID-19-related lung infection in a woman with severe hypogammaglobulinemia. Lung x-ray: **A** Extensive bilateral mixed opacities. Subpleural alveolar patches in both lungs. **B** Diffusely distributed mixed opacities with alveolar foci leading to consolidation in the left retrocardiac region. **C** Mixed opacities with bilateral patchy distribution with alveolar foci in both lower lobes and a tendency toward consolidation in the left retrocardiac region. **D** Diffuse mixed reticular and alveolar opacities, with areas of bilateral basal consolidation. Effacement of the right costophrenic angle, probably secondary to consolidation. *PaFi* arterial oxygen pressure/inspired oxygen fraction, *RT–PCR* reverse transcription-polymerase chain reaction

Based on the National Early Warning Score (NEWS) of 5 points, she was admitted to the intensive care unit (ICU), where she spent 36 days until her death. She received management for her condition with high-flow nasal cannula (HFNC) oxygen therapy, thromboprophylaxis, intravenous steroids, and empirical antibiotic therapy. Two days later, she presented with acute hypoxemic respiratory failure with clinical and radiological deterioration. Her arterial oxygen pressure/inspired oxygen fraction (PaFi) was 67.5 despite HFNC with high parameters [flow 80 L per minute and inspired oxygen fraction (FiO_2_) 0.8]. Therefore, she required mechanical ventilation. On Day 3, due to sudden respiratory deterioration, computed tomography angiography of the pulmonary arteries was performed, with no evidence of pulmonary thromboembolism. Microbiological studies of bronchoalveolar lavage (BAL) were negative. However, the respiratory molecular panel on Day 5 detected *Klebsiella pneumoniae* associated with the resistance genes *CTXM*, *Enterobacter cloacae*, and adenovirus. In addition, on Day 7, she presented with bacteremia by *E. cloacae* sensitive to ampicillin, which was treated with a targeted antibiotic. During her stay in the ICU, on Day 8, SARS-CoV-2 reverse transcription (RT)-PCR test remained positive. On Day 14, a tracheostomy was performed due to a result of prolonged orotracheal intubation, partial clinical improvement was observed, and it was possible to wean to a high-flow cannula and then to low-flow oxygen. However, again, she developed respiratory deterioration and needed invasive ventilatory support via tracheostomy. She presented with a poor neurological response despite the withdrawal of sedation. The patient did not receive antivirals for COVID, convalescent plasma, or monoclonal antibodies against SARS-Cov-2 during hospitalization. She also did not receive IGIV during hospitalization. One week before her death, IgG levels were 55 mg/dL. Brain imaging control was performed with the persistence of vasogenic edema. Finally, on Day 35, the RT-PCR test for SARS-CoV-2 remained positive, and the patient was unable to clear the SARS-CoV-2 infection and died the next day (Fig. 1).

### Genetic characterization of persistent SARS-CoV-2 viruses

Full genome sequencing of SARS-CoV-2 was performed from nasopharyngeal swab samples collected on Days 1 (first positive diagnosis for SARS-CoV-2) and 8. RNA from the samples was obtained and used for SARS-CoV-2 genomic sequencing using the ARTIC version (V3) primer set and following the LoCost V3 protocol [4] on a MinION sequencer. A third sample was obtained on Day 35, but no genomic information was available due to there being no satisfactory results in the preparation of library sequencing.

The data processing was carried out following the bioinformatics flow proposed by the ARTIC network [5]. The first sequenced SARS-CoV-2 genome (GenBank MN908947.3) was used as a reference for the assembly and assignment of single nucleotide variants. The software Medaka v.1.0.3 was also used. Genomes with a depth greater than 300X and coverage greater than 97% were obtained in both cases.

The phylogenetic analysis was carried out using the PANGOLIN software version 3.1.16 [6]. The substitutions, deletions, insertions, and reading frames of the assembled genomes were verified using Multiple Alignment using Fast Fourier Transform (MAFFT) v7.49 [7] and Nextclade v1.10.0, using Wuhan-Hu-1 as the reference genome (GenBank: MN908947.3).

From the phylogenetic analysis, both genomes were classified in the B.1.1 lineage. They have been cataloged as the European lineage with three associated characteristics: G28881G, G28882G, and G28883C. According to Genomic Surveillance Report No. 10 of the National Institute of Health (INS) of Colombia for 11 April 2021, the prevalence was 11%, which means it was one of the three lineages with the highest prevalence in the country (INS, 2021) [8].

**Fig. 2:**
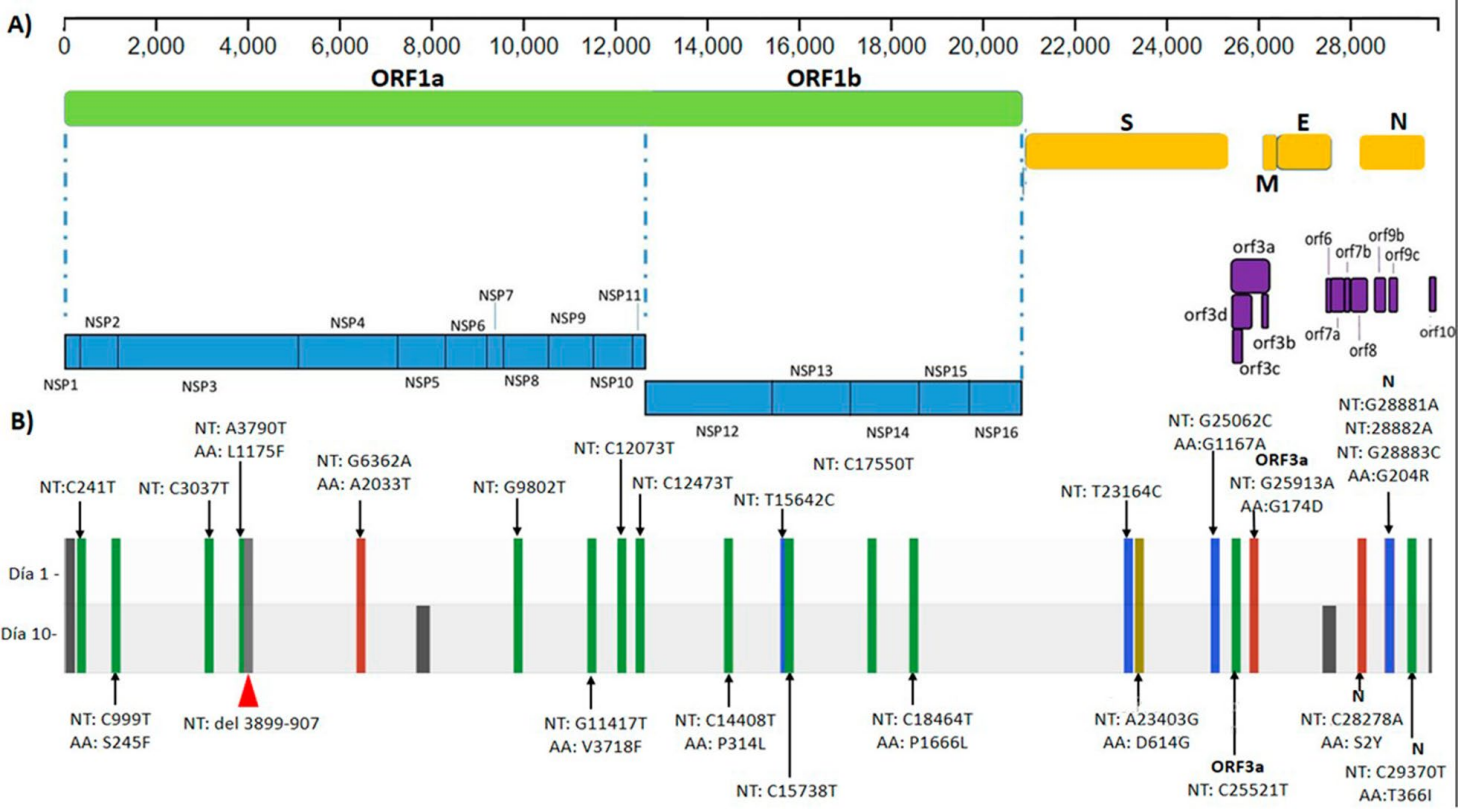
SARS-CoV-2 genomic map. **A** Consensus mutations in the two sequenced samples. **B** All nucleotide substitutions (NT) are shown. Amino acid substitutions (AAs) are indicated for all nonsynonymous substitutions. *ORF* open reading frame, *S* spike protein, *M* membrane protein E, envelope protein, *N* nucleocapsid protein, *NSP* nonstructural protein

Both genomes were identified as having 24 identical nucleotide mutations. Thirteen of these mutations resulted in nonsynonymous substitutions. Amino acid changes were demonstrated in open reading frame (ORF)1a, ORF1b, spike protein (S), ORF3a, and NSP (N) (Fig. 2A). Furthermore, a deletion of nine nucleotides (from 3899 to 907) in the *ORF1a* gene was evidenced in both genomes, which generated the three-amino-acid deletion of nonstructural protein 3 (NSP3) (Fig. 2B).

## Discussion

Patients with immunodeficiencies experience difficulty in acquiring immunity against SARS-CoV-2, which does not allow adequate clearance of the virus [1]. The prolonged survival of the virus in the host facilitates the development of variants due to various deletions and base substitutions in its genome [2]. The virus can persist several weeks before the patient can eliminate or develop symptoms of the COVID-19 virus. In some of these patients, periodic sequencing of the viral genome and the analysis of the changes that could imply the persistence of the disease and its possible interference with treatments such as convalescent plasma or monoclonal antibodies directed against protein S is performed [9–13].

The mutation rate for SARS-CoV-2 is approximately 24,000 mutations per year [14]. Limited variation within the host has been reported in SARS-CoV-2 infection. In the identified viral population, 58% of nucleotide substitutions occurred in nonstructural polyproteins (ORF1a and ORF1b), which have a multifaceted role in viral replication, transcription, morphogenesis, and evasion of the immune response. While accessory proteins are not crucial for the viral life cycle, they play an important role in viral pathogenesis and represent 8.3% of nucleotide substitutions. The mutation 14408C > T (P314 L) was observed close to the interface of the RNA-dependent RNA polymerase (RdRp) domain. Wang *et al*. [14] demonstrated that the highest frequency (10,925 times in 15,140 genotypes) of incidents occur in this domain, which is considered a hotspot of mutations. This RdRp variant has been known to not alter catalytic activity, but it can likely nullify the interaction with its cofactors and antiviral drugs. Regarding the mutations demonstrated in the *N* gene, amino acid substitutions R203K and G204R have been found among the most frequently observed in the N protein of SARS-CoV-2 [14].

The literature reported that the changes in the population dynamics of patients with persistent SARS CoV-2 infection are evident after Days 29 [9], 49 [10], and 65 [11].

In protein S, the D614G (23403A > G) mutation has been considered one of the key evolutionary changes of SARS-CoV-2. It has contributed to the worldwide transmissibility of the virus. This mutation is part of a haplotype of four mutations [including those that alter NSP12, the 5′ untranslated region (UTR), and silently NSP3], which constitutes the clade-G that originated in China and was established in Europe [13].

## Conclusions

This clinical case showed persistent SARS-CoV-2 detection after 35 days of infection in a patient with severe hypogammaglobulinemia. The sequencing of the virus showed no mutations on S protein at 8 days, indicating that, in this case, the persistence of viral detection was associated with immunodeficiency instead of changes in the viral components. Based on limited evidence, virus mutations in immunocompromised hosts require several weeks to manifest. Bacterial coinfections in our patient were present and contributed to the fatal outcome.

## Data Availability

The datasets used and/or analyzed during the current study are available from the corresponding author on reasonable request.

## Abbreviations

SARS-CoV-2: Severe acute respiratory syndrome coronavirus 2
SpO_2_: Oxygen saturation
RT-PCR: Reverse transcription-polymerase chain reaction
ICU: Intensive care unit
HFNC: High oxygen flow nasal cannula
PaFI: Arterial oxygen pressure/inspired oxygen fraction
BAL: Bronchoalveolar lavage
INS: National Institute of Health of Colombia
